# Somatic Alteration Characteristics of Early-Onset Gastric Cancer

**DOI:** 10.1155/2022/1498053

**Published:** 2022-04-22

**Authors:** Qiyang Zhou, Feng Tao, Liqing Qiu, Hanlin Chen, Hua Bao, Xue Wu, Yang Shao, Liangjie Chi, Hu Song

**Affiliations:** ^1^Jiangsu Institute of Medical Device Testing, Nanjing, Jiangsu 210012, China; ^2^Department of Gastrointestinal Surgery, Shaoxing Peopleés Hospital (Shaoxing Hospital, Zhejiang University School of Medicine, Shaoxing, Zhejiang 312000, China; ^3^Hangzhou Cancer Institution, Affiliated Hangzhou Cancer Hospital, Zhejiang University School of Medicine, Hangzhou, Zhejiang 310002, China; ^4^Geneseeq Research Institute, Nanjing Geneseeq Technology Inc., Nanjing, Jiangsu 210032, China; ^5^School of Public Health, Nanjing Medical University, Nanjing, Jiangsu, China; ^6^Department of Gastrointestinal Surgery, Shengli Clinical Medical College of Fujian Medical University, Fujian Provincial Hospital, No. 134 Dongjie, Fuzhou 350001, China; ^7^The Affilated Hospital of XuZhou Medical University, XuZhou, JiangSu 221000, China

## Abstract

Gastric cancer is one of the most common and deadly cancer types worldwide, which brings millions of dollars of economic loss each year. Patients diagnosed with early-onset gastric cancer were reported to have a worse prognosis compared to other gastric cancer patients, while the mechanisms behind such phenomenon are unknown. To identify age-dependent somatic alternations in gastric cancer, next-generation sequencing targeting 425 genes was performed on 1688 gastric tumor tissues and corresponding plasma samples. In our study, the microsatellite instability (MSI) and chromosomal instability score (CIS) values increased along with the age of patients, which indicates that older patients display a less genomic stability pattern. The differences of somatic alternations between young and old groups were compared. Somatic mutations CDH1 and copy number gains of FGFR2 were identified to enrich in the younger gastric cancer patients, which may contribute to the worse prognosis of early-onset gastric cancer patients.

## 1. Introduction

Gastric cancer (GC) is one of the most important types of cancer worldwide with a 5-year survival of 30% [1]. It is the 5th most diagnosed and 7th most prevalent cancer with an estimated number of cases of more than 1,000,000 worldwide [2]. Asia showed the highest incidence rate and mortality rate of gastric cancer, while China accounted for half of the mortality cases around the world [3]. The incidence rate and the mortality rate of gastric cancer in China were 20.6 and 15.9 per 100,000, respectively, in 2020 [4]. A large portion of gastric cancer was induced by *Helicobacter pylori* infection [5]. Gastric cancer patients with either lymphatic invasion or a tumor size >30 mm had a higher risk of lymph node metastasis [6]. Most common treatments for gastric cancer, like other types of cancers, are surgery, chemotherapy, and radiation therapy, while it was reported recently that allium vegetables may bring clinical benefit to gastric cancer treatments [7]. Currently, based on the tumor genetic sequencing results by next-generation sequencing technology, gastric cancer patients were classified into four subtypes, including Epstein–Barr virus (EBV-)-positive, microsatellite instability (MSI), chromosomal instability (CIN), and genomic stable (GS) patients. The distributions of four gastric cancer subtypes vary geographically and sexually and are age-dependent.

It is reported by several studies that gastric cancer patients diagnosed at young age normally displayed a poor prognosis. Research conducted by Ramos et al. investigated the relations between age and prognosis status in a total of 875 gastric cancer patients, including 84 young patients and 791 old patients at the age cutoff of 45. Younger patients were detected to have worse prognoses compared to older patients [8]. This result was further validated by the study led by Cheng et al., based upon the study cohort consisting of 1131 gastric cancer patients [9]. Patients at a younger age when diagnosed showed a worse prognosis pattern in the survival analysis. It was also discovered that younger gastric cancer patients were more commonly have advanced nodal and distant metastatic cancer than older patients, which may result in a more aggressive and progressive disease condition [10, 11]. However, the molecular mechanisms under these conditions are still unknown.

The molecular mechanisms behind the poor prognosis of early-onset gastric cancer may be related to somatic alteration profiles. There were a few reported genome-wide analysis studies investigating the molecular mechanisms of early-onset gastric cancer; however, most studies only focused on somatic mutations. Here, we report a large-scale study focusing on the differences of somatic alternations, including somatic mutations, copy number variations, and arm copy number variations, between young and old gastric cancer populations with the aim of identifying young-enriched somatic alternations, which may result in more aggressive gastric cancer.

## 2. Materials and Methods

### 2.1. Study Cohort

Primary gastric cancer tissue samples from 1703 patients were sequenced by a customized targeted sequencing panel. In-house quality control workflows were implemented, including FFPE damage, contamination, and matched normal control tests, to ensure the solidity of the data. At least one somatic alteration (somatic mutation or CNV) was detected in all samples sequenced. Written informed consent was collected from each patient upon sample collection according to the protocols approved by the ethical committee of their respective hospitals. 15 patients were excluded from the following analysis due to the lack of age information. There are a total of 1380 patients equal to or above 45 years old, while the rest of 308 patients were under 45 years old (Supplementary Table 1).

### 2.2. DNA Library Preparation

QIAamp DNA FFPE Tissue Kit and DNeasy Blood and Tissue Kit (Qiagen, Hilden, Germany) were applied to extract genomic DNAs in formalin-fixed paraffin-embedded (FFPE) tissues and blood control samples. The quantification process was executed by Qubit 3.0 using the dsDNA HS Assay Kit (ThermoFisher Scientific, Waltham, MA). DNA Library preparation was processed on KAPA Hyper Prep Kit (KAPA Biosystems, Wilmington, MS). A custom-made panel targeting 425 oncogenic-related genes (Geneseeq Technology Inc.) was used for hybridization enrichment (Supplementary Table 4). Following manual instructions, the capture reaction was handled by Dynabeads M-270 (Life Technologies, Carlsbad, CA, USA) and xGen Lockdown. Using Illumina p5 (5′ AAT GAT ACG GCG ACC ACC GA 3′) and p7 primers (5′ CAA GCA GAA GAC GGC ATA CGA GAT 3′), the obtained libraries were PCR-amplified in KAPA HiFi HotStart ReadyMix (KAPA Biosystems) on bead, with purification on Agencourt AMPure XP beads (Beckman Coulter) following. The library quantification by qPCR was operated in the KAPA Library Quantification Kit (KAPA Biosystems). Bioanalyzer 2100 (Agilent Technologies, Santa Clara, CA, USA) was adopted to determine the library fragment size. At last, the target-amplified library was sequenced by HiSeq4000 NGS platforms (Illumina, San Diego, CA, USA) following the producer's directions. Tumor purity was calculated by “FACETS,” and all our samples had a sample purity larger than 0.2 (Table S5).

### 2.3. Mutation Calling, MSI, TMB, CNV, and CIS Calculation

“Trimmomatic” was applied for quality control where low-quality reads (quality <20) or N bases in pair-end reads were removed. Alignment to the reference human genome (Human Genome version 19) was proceeded using “Burrows-Wheeler Aligner” (BWA) with default parameters. PCR duplicates were removed with “Picard” V2.9.4 (Broad Institute). “Genome Analysis Toolkit” (GATK 3.4.0) and “MuTect2” were chosen for local realignments around indels plus base score recalibration and somatic single-nucleotide variants (SNVs) calling, respectively. “SCALPEL” was used for small insertions/deletions (indels) calling. Mutation annotation was performed with “vcf2maf”. Recurrent sequencing errors were removed based on an error list generated from more than 500 sequencing results (mean sequencing depth >700×) on the same sequencing platform. Variants with more than three mutant reads and 1% VAF recognized in more than 10% of ordinary samples were filtered out as artifacts. Variants detected in the repeat masked regions were filtered out as well. At last, only variants in the hotpot COSMIC mutation list with more than three reads and 1% VAF, and variants with more than five reads and 2% VAF, were kept.

“ANNOVAR” [12] for variant annotation and “SIFT” [13] and “PolyPhen” [14] for protein impact prediction were performed. MSI was defined as a sample that displayed an unsteady status (relative to control samples) on more than 40% of 52 indel sites (Geneseeq Prime panel). The panel was validated for MSI status with 90 samples using “promega MSI analysis system v1.2” as a reference. The panel reached an accuracy of 95.6% (sensitivity: 96.8%; specificity: 94.9%). The cutoff of MSI was determined by the ROC curve generated by the assay validation. TMB was identified as the total number of somatic mutations detected in one sample (excluding known driver mutations). The log2 depth ratio threshold for identifying gene copy number variations (CNVs) was ±0.6. The mean percentage of genes with abnormal (log2 depth ratio >±0.2) copy numbers, weighted on 22 autosomal chromosomes, was defined as chromosomal instability score (CIS). All variant calling, CNV, MSI, and TMB definitions were validated with CLIA/CAP accreditation.

### 2.4. Viral and Bacterial Reads Identification

Reads that aligned to the human genome (hg19), mitochondrial genomes, or bacterial plasmids (NCBI RefSeq database, accessed on July 19, 2018) were filtered out from the further analysis. The k-mers algorithm of “Kraken” was applied to align the rest of the reads to NCBI microbial reference genome databases. “Bracken” (Bayesian reestimation of abundance with KrakEN) with the default settings was applied to evaluate the relative abundance of bacteria at the species or genus levels. Only samples with a high abundance of EBV reads were considered EBV-positive. The panel was validated for EBV detection using our inner data sets. The percentage of EBV-positive patients in our cohort aligns with previous studies [15, 16]. Further details about the study cohort and sample processing protocols can be found in our previous report [17].

### 2.5. Statistical Analysis

Patients under 45 years old at the time of diagnosing were identified as young patients, while patients who were or above 45 years old were classified in the old patient group. The differences in numerical variables were compared using the Chi-square test or Fisher's exact test implemented in R. Age-related trend analysis of TMB and CIS was processed using the Cuzick's trend test in “PMCMRplus” package in R. *p* values in multiple comparisons were FDR (false discovery rate) adjusted. Tests with *p* values less than 0.05 or FDR less than 0.1 were considered statistically significant.

The logistic regression algorithm “glm” in the R package “stats” was chosen to identify the differences in somatic alternations (somatic mutations, CNVs, arm CNVs) between young and old groups. As there was a large difference in sex composition between young and old groups, sex was also added as an independent variable to avoid bias. “SomaticSignatures” in the “BiocManager” package in R was applied to capture the mutation signatures of two groups, while “SomaticCancerAlterations” and “BSgenome.Hsapiens.1000genomes.hs37d5” in the “BiocManager” package were used as mutation and genome references, as recommended in the manual. All statistical analyses were performed in R (v.4.1.0).

## 3. Results

### 3.1. Study Cohorts and Clinical Characteristics

**Figure 1:**
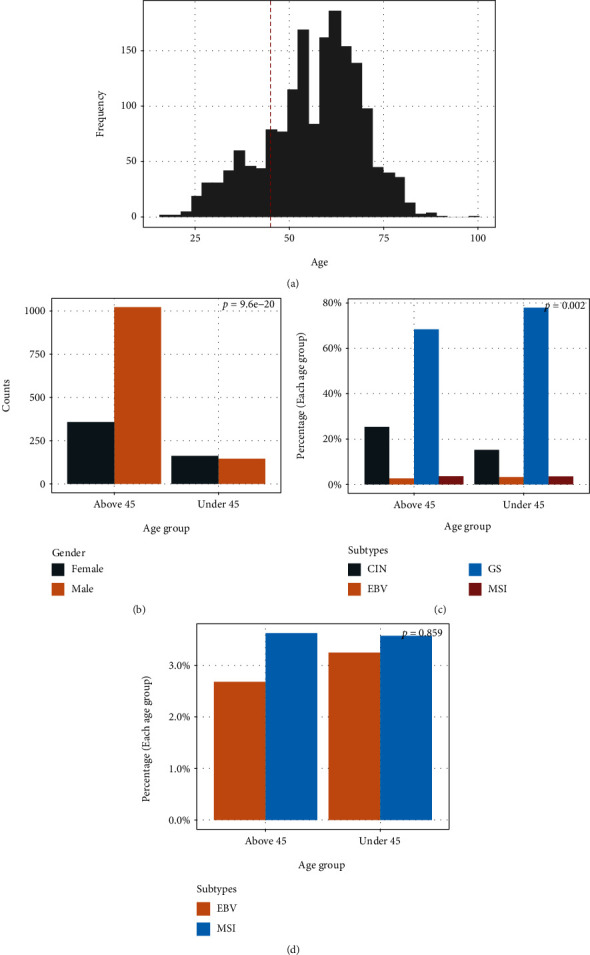
Clinical details at each age group in gastric cancer patients. (a) Number of patients in different age groups. The red vertical line denotes the age cutoff of young and old gastric cancer patients. (b) Sex distribution in young and old gastric cancer patient groups. (c) Subtype distribution in young and old gastric cancer patient groups. (d) EBV and MSI subtype distribution in young and old gastric cancer patient groups.

The sequencing data of 1688 patients with complete clinical information was analyzed. Age frequencies peaked at around 60 years old (Figure 1(a)), and the cutoff of young and old patients in our study was 45 years old based on previous studies and our sample numbers [8, 10, 11]. Among our eligible study population, 18.25% (*n* = 308) were classified as young patients, and the remaining 81.75% of patients (*n* = 1380) were classified as old patients. Significant sex disparities were observed between the two groups (*p* < 0.001), as the portion of male patients in the elder group was much higher than the younger group (Figure 1(b)).

All patients were classified into four different subtypes (EBV, MSI, CIN, GS) based on the discrimination protocols outlined in our previous study [17]. Patients with tissue samples where any EBV sequence was detected were categorized into the EBV-positive group. Among the rest of patients, patients with MSI scores equal to or higher than 0.4 were classified into the MSI group; patients with CIS values equal to or higher than 0.25 were classified into the CIN group. Patients who failed to meet all the above standards were considered GS patients. The overall subtype distribution was significantly related to age groups (*p* = 0.002), while the compositions of EBV and MSI groups in the two age groups stay consistent (Figures 1(c) and 1(d)). Higher genomic instability of older people may be the reason why the old group had more CIN patients and fewer GS patients.

### 3.2. Older GC Patients Showed a Higher Genomic Instability Pattern

**Figure 2:**
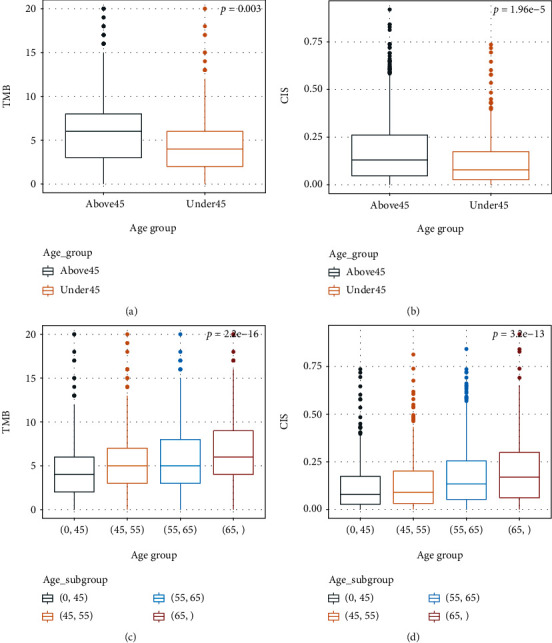
Age-dependent trends of TMB and CIS values in gastric cancer patients. *p* values represent the trend in changes across different age groups. (a) Boxplot of TMB values of young and old gastric cancer patients. (b) Boxplot of CIS values of young and old gastric cancer patients. (c) Boxplot of TMB values in different age groups. (d) Boxplot of CIS values in different age groups.

To explore the relationship between genomic instability and age, tumor mutation burden (TMB) and CIS values were compared among different age groups in the study. TMB was identified as the total number of somatic mutations of one sample, and CIS values were defined as described above. The logistic regression algorithm “glm” was applied. CIS and TMB values were passed into the binary classification model separately as numerical variables to predict the patients' age groups (with 45 years old cutoff). As mentioned above, sex was also added as a categorical variable to avoid bias. Both CIS and TMB values displayed a strong correlation with age groups and significantly enrich in the old group (FDR = 0.003 for TMB, FDR<0.001 for CIS). Both CIS and TMB showed higher value distributions in patients equal to or above 45 years old compared to patients below 45 (Figures 2(a) and 2(c)). To further validate our result, we separated patients into four age groups, which are under 45, 45 to 54, 55 to 64, 65, and older, respectively. Both CIS and TMB values increase as age grows (Figures 2(b) and 2(d)).

### 3.3. Markedly Significant Somatic Alternations Enriched in Young and Old GC Patients

**Figure 3:**
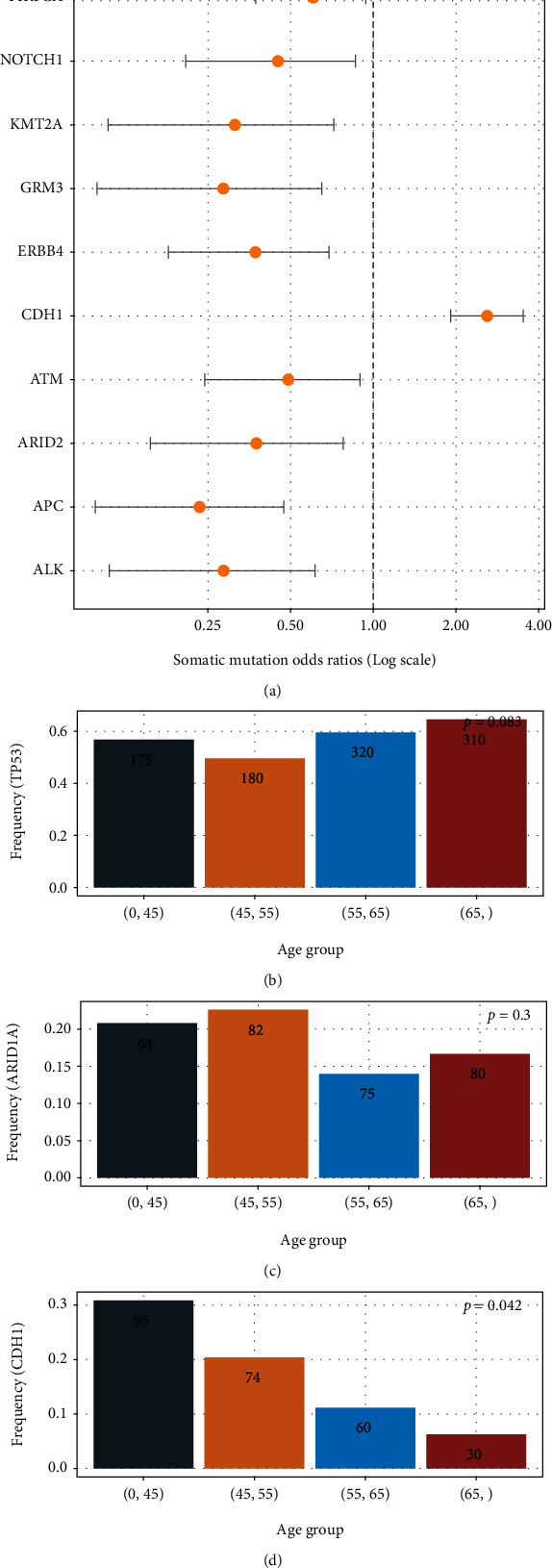
Logistic regression analysis of different somatic mutations and frequencies of different somatic mutations in multiple age groups. (a) Logistic regression analysis of different somatic mutations in gastric cancer patients. Odds ratios (ORs) represent the risk of detecting the somatic mutation when diagnosed at a young age. (b) Bar plot of somatic TP53 mutation frequencies in different age groups. (c) Bar plot of somatic ARID1A mutation frequencies in different age groups. (d) Bar plot of somatic CDH1 mutation frequencies in different age groups.

To identify somatic alternations (including somatic mutations, CNVs, arm CNVs) that related to early-onset gastric cancer, further analysis was performed between young and old gastric cancer patients. The logistic regression algorithm “glm” was used to examine somatic alternations that enrich the young GC patients. Only somatic alternations existing in more than 5% population of at least one age group were considered in the analysis. An FDR less than 0.1 was considered statistically significant. Odds ratios (ORs) were defined as the risk of a specific somatic alternation happening in young gastric cancer patients (Supplementary Table 3). A total of 11 genes whose somatic mutations were identified to be related to age, including PKHD1, PIK3CA, NOTCH1, KMT2A, GRM3, ERBB4, CDH1, ATM, ARID2, APC, and ALK (Figure 3(a)). Only CDH1 are enriched in the young group, while others are enriched in the old group. To further validate that young gastric cancer patients have higher chances of getting CDH1 somatic mutations, together with the two most abundant somatic mutations in our cohort TP53 and ARID1A, an age stratification analysis using the four age groups mentioned in the above section was applied upon these three genes. While there were no obvious changes in TP53 and ARID1A somatic mutation rates among age groups, CDH1 somatic mutation rates decreased (*p* = 0.042) as age increased (Figures 3(b)–3(d)).

**Figure 4:**
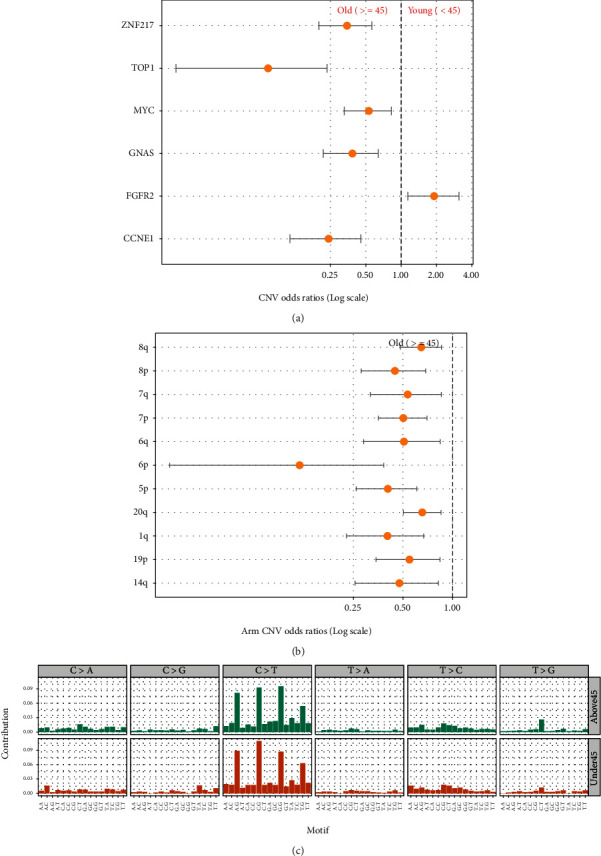
Logistic regression analysis of different somatic copy number variations (CNVs) and arm copy number variations (arm CNVs), and somatic mutation signature analysis of young and age gastric cancer groups. (a) Logistic regression analysis of different somatic CNVs in gastric cancer patients. (b) Logistic regression analysis of different arm CNVs in gastric cancer patients. (c) Somatic mutation signature patterns of young and old gastric cancer patients.

**Figure 5:**
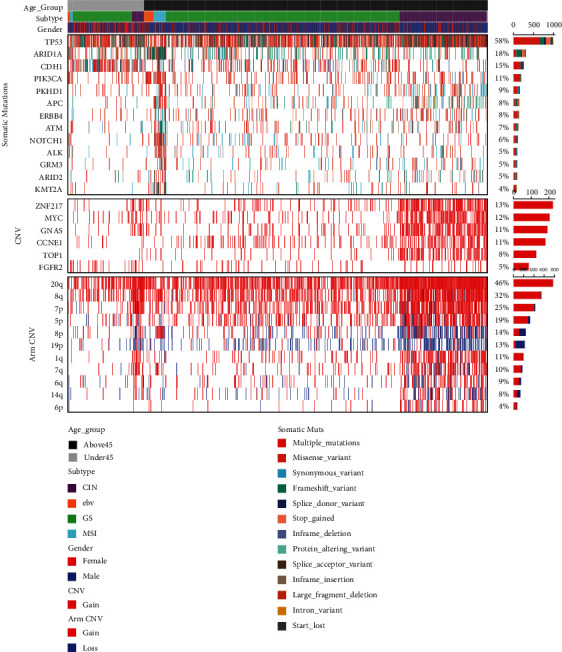
Oncoprint of most frequent somatic mutations (TP53, ARID1A) in gastric cancer patients and significant somatic alterations in logistic regression analysis.

There were a total of six age-dependent CNVs identified, including ZNF217, TOP1, MYC, GNAS, FGFR2, and CCNE1 (Figure 4(a)). Only FGFR2 enriched in the young gastric cancer patients (FDR = 0.022). All 11 arm CNVs significantly related to age were enriched in the old group (Figure 4(b)). All significant somatic alteration patterns are presented in Figure 5. The distribution of significant somatic alterations between two age groups can be seen in Supplementary Table 2.

### 3.4. Somatic Signatures and Germline Mutations

To explore whether there were any differences in somatic signatures between old and young gastric cancer patients, the “SomaticSignatures” package was applied to depict 96-motif somatic signatures patterns. No marked difference in mutation signatures between the two groups was observed (Figure 4(c)). None of the germline mutations detected in all 1688 GC patients passed the non-specific filtering threshold.

In this study, we revealed the somatic alteration characteristics of early-onset gastric cancer. Sex was identified as an important risk factor for gastric cancer, which males suffered from a higher risk of gastric cancer13. Based on the result of our study, the ratio of male and female gastric cancer patients was near 1 in the younger group, while, in the older group, male patients were almost three times as many as female patients. This was consistent with the previous report that sex disparity was negligible under 45 and maximized at around 6514. Old gastric cancer patients also suffered from higher genomic instability, based on the CIS and TMB values and the MSI subtype ratio that increased with age. Genomic Instability, which may cause functional decline and disease, was known to associate with aging15. However, there was no difference recognized in somatic mutation signatures between young and old groups.

## 4. Discussion

In this study, we revealed the somatic alteration characteristics of early-onset gastric cancer. Sex was identified as an important risk factor for gastric cancer, which males suffered from a higher risk of gastric cancer [18]. Based on the result of our study, the ratio of male and female gastric cancer patients was near 1 in the younger group, while in the older group, male patients were almost three times as many as female patients. This was consistent with the previous report that sex disparity was negligible under 45 and maximized at around 65 [19]. Old gastric cancer patients also suffered from higher genomic instability, based on the CIS and TMB values and the MSI subtype ratio that increased with age. Genomic instability, which may cause functional decline and disease, was known to associate with aging [20]. However, there was no difference recognized in somatic mutation signatures between young and old groups.

As older patients had a more unstable genomic profile, the frequencies of somatic alterations were supposed to increase along with the age. Hence, the somatic alterations which have higher happening rates in early-onset gastric cancer may be the reason for the prognosis difference between old and young gastric cancer patients. In our study, somatic CDH1 mutations and FGFR2 CNV gains were identified to enrich in the younger gastric cancer patients. It is reported by multiple studies that germline CDH1 mutations were verified to play an important role in hereditary diffuse gastric cancer (HDGC). Over 25% of HDGC patients and more than 67% of early-onset HDGC patients were reported to carry germline CDH1 mutations [21, 22]. CDH1 was known as a tumor suppressor, and germline mutations may inactivate CDH1, which will result in tumor progression and migration. However, the study of somatic CDH1 mutations and early-onset gastric cancer was little. Some large-scale genomic analyses validated our result. Cho et al. compared the genomic profiles of 109 early-onset and 115 late-onset gastric cancer patients [23], while Setia et al. analyzed the mutation patterns of 81 early-onset gastric cancer patients and 975 all-age-range cBioPortal gastric cancer patients [24]. They all confirmed that a higher rate of somatic CDH1 mutations was observed in early-onset gastric cancer patients, while our large cohort study further consolidates this phenomenon. It is interesting that the frequencies of somatic CDH1 mutations significantly decrease along with age. FGFR2 was an interesting potential therapeutic target of gastric cancer. FGFR2 was identified as a protumor gene, and the FGFR2 CNV gains may result in tumor proliferation. There were several studies revealed that the FGFR2 markedly overexpressed in gastric cancer tissues [25, 26]. Here, our study first reported the relationship between FGFR2 overexpression and early-onset gastric cancer.

The lack of clinicopathologic features in this analysis, which is a potential limitation of our study, confined us from investigating the relationships among clinicopathologic features, somatic alterations, and early-onset gastric cancer. However, there are several previous research exploring the difference of clinicopathological features between early- and late-onset gastric cancer patients. A study conducted by Yukiko et al. compared clinical features of 136 young gastric cancer patients under 40 to 1435 old patients from 60 to 69 [27]. Younger patients experienced fewer comorbidities and postoperative complications, together with more lymph node metastasis events, which is a strong risk factor for cancer relapse. Research by Taro et al. identified macroscopic type, depth of invasion, and distant metastasis as independent prognosis factors of young gastric cancer patients using a cohort consisting of 169 young patients (under 40) and 3649 old patients (above 40) [28]. Nevertheless, the research examining the relationship between somatic alternations and clinicopathologic factors of early-onset gastric cancer is still lacking.

These findings of our study suggest that the somatic CDH1 mutations and FGFR2 copy number gains may play an important role in gastric cancer development, while their higher frequencies in younger patients may contribute to worsening prognosis consequences. FGFR2 was first reported to relate to early-onset gastric cancer. Somatic CDH1 mutations and FGFR2 copy number gains both can facilitate cancer progress and result in more aggressive oncology conditions. Further studies investigating the mechanisms behind such phenomenon could contribute to better understandings and treatment developments for early-onset gastric cancer. Studies exploring the link between somatic alternations and clinicopathological features of early-onset gastric cancer are promising.

## Data Availability

The data sets used and/or analyzed in the current study are available from the corresponding author on reasonable request. Certain restrictions may apply.

## Supplementary Materials

Supplementary Materials Table S1. Clinical characteristics of young(<45 years) and old(≥45 years) gastric cancer patients. Table S2. Distribution of significant somatic alternations in logistic regression analysis. Table S3. Logistic regression analysis results of significant somatic alternations. Table S4. Gene list of the Geneseeq Prime™ 425-gene panel. Table S5. Tumor purity of the study cohort.

## References

[1] Ferlay J., Soerjomataram I., Dikshit R. (2015). Cancer incidence and mortality worldwide: sources, methods and major patterns in GLOBOCAN 2012. *International Journal of Cancer*.

[2] Rawla P., Barsouk A. (2019). Epidemiology of gastric cancer: global trends, risk factors and prevention. *Przeglad gastroenterologiczny*.

[3] Chen W., Zheng R., Baade P. D. (2016). Cancer statistics in China, 2015. *CA: a Cancer Journal for Clinicians*.

[4] IARC (2020). *Estimated age-standardized incidence rates (world) in 2020, stomach, both sexes, all ages*.

[5] Charitos I. A., D’Agostino D., Topi S., Bottalico L. (2021). 40 years of helicobacter pylori: a revolution in biomedical thought. *Gastroenterol. Insights*.

[6] Nagao K., Ebi M., Shimura T. (2022). The modified eCura system for identifying high-risk lymph node metastasis in patients with early gastric cancer resected by endoscopic submucosal dissection. *Gastroenterol. Insights*.

[7] Forma A., Chilimoniuk Z., Januszewski J., Sitarz R. (2021). The potential application of allium extracts in the treatment of gastrointestinal cancers. *Gastroenterol. Insights*.

[8] Ramos M. F. K. P., Pereira M. A., Sagae V. M. T. (2019). Gastric cancer in young adults: a worse prognosis group?. *Revista do Colégio Brasileiro de Cirurgiões*.

[9] Cheng L., Chen S., Wu W. (2020). Gastric cancer in young patients: a separate entity with aggressive features and poor prognosis. *Journal of Cancer Research and Clinical Oncology*.

[10] Moore A., Hikri E., Goshen-Lago T. (2020). Young-onset gastric cancer and Epstein-Barr virus (EBV) - a major player in the pathogenesis?. *BMC Cancer*.

[11] Al-Refaie W. B., Hu C. Y., Pisters P. W., Chang G. J. (2011). Gastric adenocarcinoma in young patients: a population-based appraisal. *Annals of Surgical Oncology*.

[12] Wang K., Li M., Hakonarson H. (2010). ANNOVAR: functional annotation of genetic variants from high-throughput sequencing data. *Nucleic Acids Research*.

[13] Ng P. C., Henikoff S. (2003). SIFT: predicting amino acid changes that affect protein function. *Nucleic Acids Research*.

[14] Adzhubei I., Jordan D. M., Sunyaev S. R. (2013). Predicting functional effect of human missense mutations using PolyPhen-2. *Current Protocols in Human Genetics*.

[15] Truong C. D., Feng W., Li W. (2009). Characteristics of Epstein-Barr virus-associated gastric cancer: a study of 235 cases at a comprehensive cancer center in U.S.A. *Journal of Experimental & Clinical Cancer Research*.

[16] Koriyama C., Akiba S., Minakami Y., Eizuru Y. (2005). Environmental factors related to Epstein-Barr virus-associated gastric cancer in Japan. *Journal of Experimental & Clinical Cancer Research*.

[17] Zhang X., Liu F., Bao H. (2021). Distinct genomic profile in h. pylori-associated gastric cancer. *Cancer Medicine*.

[18] Cavatorta O., Scida S., Miraglia C. (2018). Epidemiology of gastric cancer and risk factors. *Acta Bio-Medica*.

[19] Lou L., Wang L., Zhang Y. (2020). Sex difference in incidence of gastric cancer: an international comparative study based on the global burden of disease study 2017. *BMJ Open*.

[20] Vijg J., Suh Y. (2013). Genome instability and aging. *Annual Review of Physiology*.

[21] Luo W., Fedda F., Lynch P., Tan D. (2018). CDH1 gene and hereditary diffuse gastric cancer syndrome: molecular and histological alterations and implications for diagnosis and treatment. *Frontiers in Pharmacology*.

[22] Hakkaart C., Ellison-Loschmann L., Day R. (2019). Germline CDH1 mutations are a significant contributor to the high frequency of early-onset diffuse gastric cancer cases in New Zealand Māori. *Familial Cancer*.

[23] Cho S. Y., Park J. W., Liu Y. (2017). Sporadic early-onset diffuse gastric cancers have high frequency of somatic CDH1 Alterations, but low frequency of somatic RHOA mutations compared with late-onset cancers. *Gastroenterology*.

[24] Setia N., Wang C. X., Lager A. (2020). Morphologic and molecular analysis of early-onset gastric cancer. *Cancer*.

[25] Huang T., Liu D., Wang Y. (2018). FGFR2 promotes gastric cancer progression by inhibiting the expression of thrombospondin4 via PI3K-Akt-Mtor pathway. *Cellular Physiology and Biochemistry*.

[26] Zhang J., Wong C. C., Leung K. T. (2020). FGF18-FGFR2 signaling triggers the activation of c-Jun-YAP1 axis to promote carcinogenesis in a subgroup of gastric cancer patients and indicates translational potential. *Oncogene*.

[27] Takatsu Y., Hiki N., Nunobe S. (2016). Clinicopathological features of gastric cancer in young patients. *Gastric Cancer*.

[28] Isobe T., Hashimoto K., Kizaki J. (2013). Characteristics and prognosis of gastric cancer in young patients. *Oncology Reports*.

