# Supplementary material for: Somatic Alteration Characteristics of Early-Onset Gastric Cancer
Source: J Oncol. 2022 Apr 22;2022:1498053. doi: 10.1155/2022/1498053 (PMC9054482; doi:10.1155/2022/1498053)
Supplement: Supplementary Materials — Table S1. Clinical characteristics of young(<45 years) and old(≥45 years) gastric cancer patients. Table S2. Distribution of significant somatic alternations in logistic regression analysis. Table S3. Logistic regression analysis results of significant somatic alternations. Table S4. Gene list of the Geneseeq Prime™ 425-gene panel. Table S5. Tumor purity of the study cohort. [file 1498053.f1.docx]

**Table S1**

**Clinical characteristics of young(<45 years) and old(≥45 years) gastric cancer patients**

| **Clinical characteristics** | | **Age < 45 (n, frequency [%])** | | **Age ≥ 45 (n, frequency [%])** | | **Total** |
| --- | --- | --- | --- | --- | --- | --- |
|  |  |  |  |  |  |  |
| **Sex** | Male | 146 | 47.40% | 1022 | 74% | 1168 |
|  | Female | 162 | 52.60% | 358 | 25.90% | 520 |
| **Subgroup** | CIN | 47 | 15.30% | 350 | 25.40% | 397 |
|  | EBV | 10 | 3.20% | 37 | 2.70% | 47 |
|  | GS | 240 | 77.90% | 943 | 68.30% | 1183 |
|  | MSI | 11 | 3.60% | 50 | 3.60% | 61 |

**Table S2**

**Distribution of significant somatic alternations in logistic regression analysis**

|  | **Age < 45 (308)**  **(n, frequency [%])** | **Age ≥ 45 (1380)**  **(n, frequency [%])** |
| --- | --- | --- |
| **Somatic Mutations** | | |
| **PKHD1** | 15 4.9% | 133 9.6% |
| **PIK3CA*** | 24 7.8% | 155 11.2% |
| **NOTCH1*** | 9 2.9% | 99 7.2% |
| **KMT2A*** | 5 1.6% | 70 5.1% |
| **GRM3** | 5 1.6% | 78 5.7% |
| **ERBB4** | 10 3.2% | 121 8.8% |
| **CDH1** | 95 30.8% | 164 11.9% |
| **ATM*** | 11 3.6% | 102 7.4% |
| **ARID2** | 7 2.3% | 76 5.5% |
| **APC** | 7 2.3% | 131 9.5% |
| **ALK*** | 6 1.9% | 85 6.2% |
| **CNVs** | | |
| **ZNF217** | 17 5.5% | 206 14.9% |
| **TOP1** | 2 0.6% | 127 9.2% |
| **MYC*** | 23 7.5% | 181 13.1% |
| **GNAS** | 16 5.2% | 176 12.8% |
| **FGFR2*** | 25 8.1% | 61 4.4% |
| **CCNE1** | 9 2.9% | 172 12.5% |
| **Arm CNVs** | | |
| **8q** | 77 25.0% | 471 34.1% |
| **8p** | 24 7.8% | 217 15.7% |
| **7q** | 20 6.5% | 155 11.2% |
| **7p** | 47 15.3% | 379 27.5% |
| **6q** | 17 5.5% | 132 9.6% |
| **6p** | 2 0.6% | 72 5.2% |
| **5p** | 27 8.8% | 297 21.5% |
| **20q** | 111 36.0% | 662 45.0% |
| **1q** | 16 5.2% | 178 12.9% |
| **19p** | 25 8.1% | 195 14.1% |
| **14q** | 14 4.5% | 122 8.8% |

*Labeled as actionable genes in oncology in OncoKB database

**Table S3**

**Logistic regression analysis results of significant somatic alternations**

|  | **Estimate** | **StdError** | **Z.value** | **P.value** | **OR** | **Conf2.5** | **Conf97.5** | **FDR** |
| --- | --- | --- | --- | --- | --- | --- | --- | --- |
| **Somatic Mutations** | | | | | | | | |
| **PKHD1** | -0.68 | 0.28 | -2.39 | 2.0E-2 | 0.51 | 0.28 | 0.86 | 5.7E-2 |
| **CDH1** | 0.95 | 0.15 | 6.16 | 7.5E-10 | 2.60 | 1.91 | 3.51 | 2.0E-8 |
| **GRM3** | -1.26 | 0.47 | -2.67 | 7.6E-3 | 0.28 | 0.10 | 0.65 | 4.1E-2 |
| **NOTCH1*** | -0.80 | 0.36 | -2.23 | 2.6E-2 | 0.45 | 0.21 | 0.86 | 7.7E-2 |
| **PIK3CA*** | -0.50 | 0.23 | -2.152 | 3.1E-2 | 0.60 | 0.37 | 0.94 | 7.7E-2 |
| **ERBB4** | -0.99 | 0.34 | -2.91 | 3.6E-3 | 0.37 | 0.18 | 0.69 | 2.5E-2 |
| **ATM*** | -0.71 | 0.33 | -2.17 | 3.0E-2 | 0.49 | 0.24 | 0.90 | 7.7E-2 |
| **ARID2** | -0.98 | 0.41 | -2.41 | 1.6E-2 | 0.38 | 0.15 | 0.78 | 5.7E-2 |
| **KMT2A*** | -1.16 | 0.47 | -2.45 | 1.4E-2 | 0.31 | 0.11 | 0.72 | 5.7E-2 |
| **APC** | -1.46 | 0.40 | -3.67 | 2.4E-4 | 0.23 | 0.10 | 0.47 | 3.3E-3 |
| **ALK*** | -1.25 | 0.43 | -2.90 | 3.7E-3 | 0.29 | 0.11 | 0.61 | 2.5E-2 |
| **CNVs** | | | | | | | | |
| **ZNF217** | -1.06 | 0.26 | -4.02 | 5.8E-5 | 0.35 | 0.20 | 0.56 | 3.5E-4 |
| **GNAS** | -0.96 | 0.27 | -3.50 | 4.6E-4 | 0.38 | 0.22 | 0.64 | 1.4E-3 |
| **FGFR2*** | 0.65 | 0.25 | 2.54 | 1.1E-2 | 1.91 | 1.14 | 3.11 | 2.2E-2 |
| **MYC*** | -0.63 | 0.24 | -2.69 | 7.1E-3 | 0.53 | 0.33 | 0.83 | 1.7E-2 |
| **CCNE1** | -1.42 | 0.35 | -4.05 | 5.1E-5 | 0.24 | 0.11 | 0.45 | 3.5E-4 |
| **TOP1** | -2.61 | 0.72 | -3.64 | 2.7E-4 | 0.07 | 0.01 | 0.23 | 1.1E-3 |
| **Arm CNVs** | | | | | | | | |
| **20q** | -0.42 | 0.13 | -3.18 | 1.5E-3 | 0.65 | 0.50 | 0.85 | 1.1E-2 |
| **7p** | -0.69 | 0.17 | -3.99 | 6.7E-5 | 0.50 | 0.35 | 0.70 | 1.2E-3 |
| **14q** | -0.74 | 0.29 | -2.52 | 1.1E-2 | 0.48 | 0.26 | 0.82 | 4.2E-2 |
| **6p** | -2.14 | 0.72 | -2.95 | 3.1E-3 | 0.12 | 0.02 | 0.38 | 1.7E-2 |
| **6q** | -0.68 | 0.27 | -2.51 | 1.2E-2 | 0.51 | 0.29 | 0.84 | 4.2E-2 |
| **8q** | -0.44 | 0.15 | -2.99 | 2.8E-3 | 0.64 | 0.48 | 0.86 | 1.7E-2 |
| **8p** | -0.81 | 0.23 | -3.52 | 4.3E-4 | 0.45 | 0.28 | 0.69 | 5.3E-3 |
| **5p** | -0.91 | 0.22 | -4.21 | 2.6E-5 | 0.40 | 0.26 | 0.61 | 9.5E-4 |
| **1q** | -0.91 | 0.27 | -3.34 | 8.5E-4 | 0.40 | 0.23 | 0.67 | 7.9E-3 |
| **19p** | -0.60 | 0.23 | -2.66 | 7.8E-3 | 0.55 | 0.34 | 0.84 | 3.6E-2 |
| **7q** | -0.63 | 0.25 | -2.50 | 1.2E-2 | 0.53 | 0.32 | 0.85 | 4.2E-2 |

*Labeled as actionable genes in oncology in OncoKB database

**Table S4**

**Gene list of the Geneseeq Prime™ 425-gene panel.**

| ABCB1(MDR1) | BMPR1A | CUX1 | ETV6 | GSTP1 | LYN | NKX2-1 | PPARD | SDC4 | THADA |
| --- | --- | --- | --- | --- | --- | --- | --- | --- | --- |
| ABCB4 | BRAF | CXCR4 | EWSR1 | GSTT1 | LZTR1 | NKX2-4 | PPP2R1A | SDHA | TMEM127 |
| ABCC2(MRP2) | BRCA1 | CYLD | EXT1 | HDAC2 | MAP2K1(MEK1) | NOTCH1 | PRDM1 | SDHB | TMPRSS2 |
| ADH1A | BRCA2 | CYP19A1 | EXT2 | HDAC9 | MAP2K2(MEK2) | NOTCH2 | PRF1 | SDHC | TNFAIP3 |
| ADH1B | BRD4 | CYP2A13 | EZH2 | HGF | MAP2K4 | NOTCH3 | PRKACA | SDHD | TNFRSF11A |
| ADH1C | BRIP1 | CYP2A6 | FANCA | HLA-A | MAP3K1 | NPM1 | PRKACG | SEPT9  (SEPTIN9) | TNFRSF14 |
| AIP | BTG2 | CYP2A7 | FANCC | HNF1A | MAP3K4 | NQO1 | PRKAR1A | SETBP1 | TNFRSF19 |
| AKT1 | BTK | CYP2B6*6 | FANCD2 | HNF1B | MAP4K3 | NRAS | PRKCI | SETD2 | TNFSF11 |
| AKT2 | BUB1B | CYP2C19*2 | FANCE | HRAS | MAX | NRG1 | PRKDC | SF3B1 | TOP1 |
| AKT3 | c11orf30(EMSY) | CYP2C9*3 | FANCF | HSD3B1 | MCL1 | NSD1 | PRSS1 | SGK1 | TOP2A |
| ALDH2 | CASP8 | CYP2D6 | FANCG | IDH1 | MDM2 | NTRK1 | PRSS3 | SLC34A2 | TP53 |
| ALK | CBL | CYP3A4*4 | FANCI | IDH2 | MDM4 | NTRK2 | PTCH1 | SLC3A2 | TP63 |
| AMER1 | CBLB | CYP3A5 | FANCL | IFNG | MECOM | NTRK3 | PTEN | SLC7A8 | TPMT |
| APC | CCND1 | DAXX | FANCM | IFNGR1 | MED12 | PAK3 | PTK2 | SMAD2 | TSC1 |
| AR | CCNE1 | DDR2 | FAT1 | IGF1R | MEF2B | PALB2 | PTPN11 | SMAD3 | TSC2 |
| ARAF | CD274(PD-L1) | DENND1A | FBXW7 | IGF2 | MEN1 | PALLD | PTPN13 | SMAD4 | TSHR |
| ARID1A | CD74 | DHFR | FGF19 | IKBKE | MET | PARK2(PRKN) | PTPRD | SMAD7 | TTF1 |
| ARID1B | CDA | DICER1 | FGFR1 | IKZF1 | MGMT | PARP1 | QKI | SMARCA4 | TUBB3 |
| ARID2 | CDC73 | DLL3 | FGFR2 | IL7R | MITF | PARP2 | RAC1 | SMARCB1 | TUBB4A |
| ARID5B | CDH1 | DNMT3A | FGFR3 | INPP4B | MLH1 | PAX5 | RAC3 | SMO | TUBB4B |
| ASCL4 | CDK10 | DPYD | FGFR4 | IRF2 | MLH3 | PBRM1 | RAD50 | SOS1 | TUBB6 |
| ASXL1 | CDK12 | DUSP2 | FH | JAK1 | MLLT1 | PDCD1(PD1) | RAD51 | SOX1 | TYMS |
| ATF1 | CDK4 | EGFR | FLCN | JAK2 | MLLT3 | PDCD1LG2(PD-L2) | RAD51B | SOX14 | U2AF1 |
| ATIC | CDK6 | EML4 | FLT1  (VEGFR1) | JAK3 | MLLT4(AFDN) | PDE11A | RAD51C | SOX2 | UGT1A1 |
| ATM | CDK8 | EP300 | FLT3 | JARID2 | MPL | PDGFRA | RAD51D | SOX21 | VAMP2 |
| ATR | CDKN1A | EPAS1 | FLT4 | JUN | MRE11A  (MRE11) | PDGFRB | RAD54L | SPOP | VEGFA |
| ATRX | CDKN1B | EPCAM | FOXA1 | KDM5A | MSH2 | PDK1 | RAF1 | SPRY4 | VHL |
| AURKA | CDKN1C | EPHA2 | FOXP1 | KDM6A | MSH6 | PGR | RARA | SRC | WAS |
| AURKB | CDKN2A | EPHA3 | FRG1 | KDR  (VEGFR2) | MTHFR | PHOX2B | RARG | SRY | WISP3  (CCN6) |
| AXIN2 | CDKN2B | EPHA5 | GATA1 | KEAP1 | MTOR | PIK3C3 | RASGEF1A | STAG2 | WRN |
| AXL | CDKN2C | EPHB2 | GATA2 | KIF1B | MUTYH | PIK3CA | RB1 | STAT3 | WT1 |
| B2M | CEBPA | ERBB2  (HER2) | GATA3 | KIF5B | MYC | PIK3R1 | RECQL4 | STK11 | XPA |
| BAD | CEP57 | ERBB2IP  (ERBIN) | GATA4 | KIT | MYCL | PIK3R2 | RELN | STMN1 | XPC |
| BAI3  (ADGRB3) | CHD4 | ERBB3 | GATA6 | KITLG | MYCN | PKHD1 | RET | STT3A | XRCC1 |
| BAK1 | CHEK1 | ERBB4 | GNA11 | KLLN | MYD88 | PLAG1 | RHOA | SUFU | YAP1 |
| BAP1 | CHEK2 | ERCC1 | GNAQ | KMT2A(MLL) | MYH9 | PLK1 | RICTOR | TAP1 | ZNF2 |
| BARD1 | CREBBP | ERCC2 | GNAS | KMT2B | NAT1 | PMS1 | RNF43 | TAP2 | ZNF217 |
| BAX | CRKL | ERCC3 | GRIN2A | KMT2C | NBN | PMS2 | ROS1 | TEK | ZNF703 |
| BCL2 | CSF1R | ERCC4 | GRM3 | KMT2D  (MLL2) | NCOR1 | POLD1 | RPTOR | TEKT4 |  |
| BCL2L11(BIM) | CTCF | ERCC5 | GRM8 | KRAS | NF1 | POLD3 | RRM1 | TERC |  |
| BCR | CTLA4 | ESR1 | GSTM1 | LHCGR | NF2 | POLE | RUNX1 | TERT |  |
| BIRC3 | CTNNB1 | ETV1 | GSTM4 | LMO1 | NFE2L2 | POLH | RUNX1T1 | TET2 |  |
| BLM | CUL3 | ETV4 | GSTM5 | LRP1B | NFKBIA | POT1 | SBDS | TGFBR2 |  |

**Table S5**

**Tumor Purity of the study cohort**

| **Sample** | **Purity** | **Sample** | **Purity** | **Sample** | **Purity** | **Sample** | **Purity** |
| --- | --- | --- | --- | --- | --- | --- | --- |
| **S1** | 0.23 | **S423** | 0.24 | **S845** | 0.26 | **S1267** | 0.68 |
| **S2** | 0.3 | **S424** | 0.5 | **S846** | 0.3 | **S1268** | 0.33 |
| **S3** | 0.21 | **S425** | 0.85 | **S847** | 0.23 | **S1269** | 0.36 |
| **S4** | 0.28 | **S426** | 0.28 | **S848** | 0.3 | **S1270** | 0.21 |
| **S5** | 0.26 | **S427** | 0.3 | **S849** | 0.2 | **S1271** | 0.32 |
| **S6** | 0.36 | **S428** | 0.26 | **S850** | 0.28 | **S1272** | 0.26 |
| **S7** | 0.3 | **S429** | 0.27 | **S851** | 0.86 | **S1273** | 0.21 |
| **S8** | 0.22 | **S430** | 0.3 | **S852** | 0.26 | **S1274** | 0.29 |
| **S9** | 0.4 | **S431** | 0.32 | **S853** | 0.27 | **S1275** | 0.22 |
| **S10** | 0.27 | **S432** | 0.24 | **S854** | 0.44 | **S1276** | 0.21 |
| **S11** | 0.21 | **S433** | 0.29 | **S855** | 0.37 | **S1277** | 0.3 |
| **S12** | 0.76 | **S434** | 0.29 | **S856** | 0.36 | **S1278** | 0.35 |
| **S13** | 0.3 | **S435** | 0.21 | **S857** | 0.21 | **S1279** | 0.22 |
| **S14** | 0.3 | **S436** | 0.27 | **S858** | 0.47 | **S1280** | 0.32 |
| **S15** | 0.22 | **S437** | 0.29 | **S859** | 0.28 | **S1281** | 0.23 |
| **S16** | 0.3 | **S438** | 0.49 | **S860** | 0.45 | **S1282** | 0.3 |
| **S17** | 0.31 | **S439** | 0.29 | **S861** | 0.26 | **S1283** | 0.29 |
| **S18** | 0.4 | **S440** | 0.51 | **S862** | 0.2 | **S1284** | 0.3 |
| **S19** | 0.3 | **S441** | 0.89 | **S863** | 0.3 | **S1285** | 0.5 |
| **S20** | 0.21 | **S442** | 0.24 | **S864** | 0.3 | **S1286** | 0.21 |
| **S21** | 0.28 | **S443** | 0.33 | **S865** | 0.3 | **S1287** | 0.2 |
| **S22** | 0.21 | **S444** | 0.23 | **S866** | 0.36 | **S1288** | 0.27 |
| **S23** | 0.28 | **S445** | 0.21 | **S867** | 0.59 | **S1289** | 0.25 |
| **S24** | 0.37 | **S446** | 0.29 | **S868** | 0.3 | **S1290** | 0.44 |
| **S25** | 0.23 | **S447** | 0.27 | **S869** | 0.39 | **S1291** | 0.34 |
| **S26** | 0.29 | **S448** | 0.3 | **S870** | 0.25 | **S1292** | 0.26 |
| **S27** | 0.21 | **S449** | 0.49 | **S871** | 0.2 | **S1293** | 0.29 |
| **S28** | 0.32 | **S450** | 0.39 | **S872** | 0.33 | **S1294** | 0.23 |
| **S29** | 0.42 | **S451** | 0.29 | **S873** | 0.22 | **S1295** | 0.2 |
| **S30** | 0.28 | **S452** | 0.29 | **S874** | 0.26 | **S1296** | 0.25 |
| **S31** | 0.3 | **S453** | 0.48 | **S875** | 0.28 | **S1297** | 0.23 |
| **S32** | 0.26 | **S454** | 0.27 | **S876** | 0.28 | **S1298** | 0.64 |
| **S33** | 0.24 | **S455** | 0.29 | **S877** | 0.8 | **S1299** | 0.23 |
| **S34** | 0.25 | **S456** | 0.23 | **S878** | 0.29 | **S1300** | 0.64 |
| **S35** | 0.42 | **S457** | 0.3 | **S879** | 0.24 | **S1301** | 0.3 |
| **S36** | 0.29 | **S458** | 0.25 | **S880** | 0.26 | **S1302** | 0.36 |
| **S37** | 0.23 | **S459** | 0.27 | **S881** | 0.3 | **S1303** | 0.25 |
| **S38** | 0.45 | **S460** | 0.72 | **S882** | 0.34 | **S1304** | 0.47 |
| **S39** | 0.87 | **S461** | 0.3 | **S883** | 0.3 | **S1305** | 0.54 |
| **S40** | 0.6 | **S462** | 0.3 | **S884** | 0.28 | **S1306** | 0.33 |
| **S41** | 0.3 | **S463** | 0.27 | **S885** | 0.29 | **S1307** | 0.28 |
| **S42** | 0.4 | **S464** | 0.33 | **S886** | 0.22 | **S1308** | 0.22 |
| **S43** | 0.27 | **S465** | 0.24 | **S887** | 0.3 | **S1309** | 0.29 |
| **S44** | 0.36 | **S466** | 0.64 | **S888** | 0.3 | **S1310** | 0.25 |
| **S45** | 0.27 | **S467** | 0.3 | **S889** | 0.27 | **S1311** | 0.28 |
| **S46** | 0.25 | **S468** | 0.32 | **S890** | 0.52 | **S1312** | 0.33 |
| **S47** | 0.92 | **S469** | 0.41 | **S891** | 0.49 | **S1313** | 0.41 |
| **S48** | 0.25 | **S470** | 0.21 | **S892** | 0.42 | **S1314** | 0.25 |
| **S49** | 0.74 | **S471** | 0.51 | **S893** | 0.34 | **S1315** | 0.2 |
| **S50** | 0.23 | **S472** | 0.4 | **S894** | 0.3 | **S1316** | 0.3 |
| **S51** | 0.24 | **S473** | 0.28 | **S895** | 0.28 | **S1317** | 0.56 |
| **S52** | 0.26 | **S474** | 0.22 | **S896** | 0.46 | **S1318** | 0.34 |
| **S53** | 0.33 | **S475** | 0.45 | **S897** | 0.4 | **S1319** | 0.21 |
| **S54** | 0.29 | **S476** | 0.24 | **S898** | 0.3 | **S1320** | 0.73 |
| **S55** | 0.36 | **S477** | 0.53 | **S899** | 0.33 | **S1321** | 0.28 |
| **S56** | 0.69 | **S478** | 0.4 | **S900** | 0.4 | **S1322** | 0.64 |
| **S57** | 0.3 | **S479** | 0.22 | **S901** | 0.48 | **S1323** | 0.56 |
| **S58** | 0.34 | **S480** | 0.26 | **S902** | 0.29 | **S1324** | 0.66 |
| **S59** | 0.29 | **S481** | 0.23 | **S903** | 0.76 | **S1325** | 0.58 |
| **S60** | 0.41 | **S482** | 0.22 | **S904** | 0.62 | **S1326** | 0.24 |
| **S61** | 0.22 | **S483** | 0.63 | **S905** | 0.47 | **S1327** | 0.37 |
| **S62** | 0.3 | **S484** | 0.22 | **S906** | 0.24 | **S1328** | 0.54 |
| **S63** | 0.22 | **S485** | 0.22 | **S907** | 0.23 | **S1329** | 0.28 |
| **S64** | 0.51 | **S486** | 0.22 | **S908** | 0.27 | **S1330** | 0.3 |
| **S65** | 0.29 | **S487** | 0.44 | **S909** | 0.24 | **S1331** | 0.29 |
| **S66** | 0.3 | **S488** | 0.3 | **S910** | 0.3 | **S1332** | 0.21 |
| **S67** | 0.27 | **S489** | 0.27 | **S911** | 0.43 | **S1333** | 0.51 |
| **S68** | 0.21 | **S490** | 0.64 | **S912** | 0.39 | **S1334** | 0.84 |
| **S69** | 0.64 | **S491** | 0.4 | **S913** | 0.35 | **S1335** | 0.26 |
| **S70** | 0.61 | **S492** | 0.25 | **S914** | 0.34 | **S1336** | 0.25 |
| **S71** | 0.75 | **S493** | 0.27 | **S915** | 0.3 | **S1337** | 0.41 |
| **S72** | 0.44 | **S494** | 0.3 | **S916** | 0.3 | **S1338** | 0.3 |
| **S73** | 0.43 | **S495** | 0.27 | **S917** | 0.28 | **S1339** | 0.3 |
| **S74** | 0.3 | **S496** | 0.3 | **S918** | 0.24 | **S1340** | 0.26 |
| **S75** | 0.31 | **S497** | 0.28 | **S919** | 0.28 | **S1341** | 0.33 |
| **S76** | 0.23 | **S498** | 0.28 | **S920** | 0.22 | **S1342** | 0.3 |
| **S77** | 0.3 | **S499** | 0.3 | **S921** | 0.23 | **S1343** | 0.4 |
| **S78** | 0.3 | **S500** | 0.23 | **S922** | 0.29 | **S1344** | 0.3 |
| **S79** | 0.25 | **S501** | 0.32 | **S923** | 0.31 | **S1345** | 0.24 |
| **S80** | 0.84 | **S502** | 0.41 | **S924** | 0.48 | **S1346** | 0.23 |
| **S81** | 0.28 | **S503** | 0.23 | **S925** | 0.25 | **S1347** | 0.27 |
| **S82** | 0.39 | **S504** | 0.24 | **S926** | 0.3 | **S1348** | 0.91 |
| **S83** | 0.3 | **S505** | 0.32 | **S927** | 0.3 | **S1349** | 0.53 |
| **S84** | 0.26 | **S506** | 0.3 | **S928** | 0.28 | **S1350** | 0.22 |
| **S85** | 0.3 | **S507** | 0.27 | **S929** | 0.23 | **S1351** | 0.28 |
| **S86** | 0.28 | **S508** | 0.23 | **S930** | 0.27 | **S1352** | 0.26 |
| **S87** | 0.39 | **S509** | 0.3 | **S931** | 0.36 | **S1353** | 0.3 |
| **S88** | 0.32 | **S510** | 0.51 | **S932** | 0.32 | **S1354** | 0.22 |
| **S89** | 0.38 | **S511** | 0.26 | **S933** | 0.26 | **S1355** | 0.22 |
| **S90** | 0.3 | **S512** | 0.21 | **S934** | 0.27 | **S1356** | 0.4 |
| **S91** | 0.3 | **S513** | 0.23 | **S935** | 0.55 | **S1357** | 0.28 |
| **S92** | 0.64 | **S514** | 0.38 | **S936** | 0.44 | **S1358** | 0.25 |
| **S93** | 0.23 | **S515** | 0.21 | **S937** | 0.23 | **S1359** | 0.3 |
| **S94** | 0.32 | **S516** | 0.45 | **S938** | 0.37 | **S1360** | 0.24 |
| **S95** | 0.3 | **S517** | 0.34 | **S939** | 0.26 | **S1361** | 0.3 |
| **S96** | 0.43 | **S518** | 0.3 | **S940** | 0.3 | **S1362** | 0.24 |
| **S97** | 0.36 | **S519** | 0.55 | **S941** | 0.27 | **S1363** | 0.3 |
| **S98** | 0.38 | **S520** | 0.62 | **S942** | 0.29 | **S1364** | 0.3 |
| **S99** | 0.3 | **S521** | 0.26 | **S943** | 0.55 | **S1365** | 0.35 |
| **S100** | 0.49 | **S522** | 0.23 | **S944** | 0.62 | **S1366** | 0.27 |
| **S101** | 0.29 | **S523** | 0.23 | **S945** | 0.26 | **S1367** | 0.51 |
| **S102** | 0.22 | **S524** | 0.31 | **S946** | 0.3 | **S1368** | 0.2 |
| **S103** | 0.21 | **S525** | 0.39 | **S947** | 0.29 | **S1369** | 0.29 |
| **S104** | 0.56 | **S526** | 0.2 | **S948** | 0.34 | **S1370** | 0.2 |
| **S105** | 0.41 | **S527** | 0.3 | **S949** | 0.4 | **S1371** | 0.38 |
| **S106** | 0.31 | **S528** | 0.21 | **S950** | 0.33 | **S1372** | 0.22 |
| **S107** | 0.2 | **S529** | 0.48 | **S951** | 0.24 | **S1373** | 0.27 |
| **S108** | 0.3 | **S530** | 0.73 | **S952** | 0.22 | **S1374** | 0.3 |
| **S109** | 0.29 | **S531** | 0.68 | **S953** | 0.33 | **S1375** | 0.3 |
| **S110** | 0.3 | **S532** | 0.34 | **S954** | 0.28 | **S1376** | 0.3 |
| **S111** | 0.23 | **S533** | 0.37 | **S955** | 0.3 | **S1377** | 0.64 |
| **S112** | 0.54 | **S534** | 0.91 | **S956** | 0.74 | **S1378** | 0.3 |
| **S113** | 0.35 | **S535** | 0.36 | **S957** | 0.3 | **S1379** | 0.42 |
| **S114** | 0.27 | **S536** | 0.3 | **S958** | 0.21 | **S1380** | 0.27 |
| **S115** | 0.27 | **S537** | 0.26 | **S959** | 0.29 | **S1381** | 0.29 |
| **S116** | 0.25 | **S538** | 0.39 | **S960** | 0.38 | **S1382** | 0.54 |
| **S117** | 0.3 | **S539** | 0.27 | **S961** | 0.3 | **S1383** | 0.24 |
| **S118** | 0.26 | **S540** | 0.32 | **S962** | 0.3 | **S1384** | 0.29 |
| **S119** | 0.26 | **S541** | 0.28 | **S963** | 0.3 | **S1385** | 0.26 |
| **S120** | 0.29 | **S542** | 0.39 | **S964** | 0.29 | **S1386** | 0.26 |
| **S121** | 0.27 | **S543** | 0.31 | **S965** | 0.26 | **S1387** | 0.2 |
| **S122** | 0.21 | **S544** | 0.23 | **S966** | 0.26 | **S1388** | 0.23 |
| **S123** | 0.36 | **S545** | 0.25 | **S967** | 0.21 | **S1389** | 0.3 |
| **S124** | 0.91 | **S546** | 0.3 | **S968** | 0.3 | **S1390** | 0.21 |
| **S125** | 0.23 | **S547** | 0.34 | **S969** | 0.32 | **S1391** | 0.24 |
| **S126** | 0.25 | **S548** | 0.28 | **S970** | 0.28 | **S1392** | 0.73 |
| **S127** | 0.22 | **S549** | 0.2 | **S971** | 0.92 | **S1393** | 0.23 |
| **S128** | 0.28 | **S550** | 0.37 | **S972** | 0.6 | **S1394** | 0.3 |
| **S129** | 0.29 | **S551** | 0.23 | **S973** | 0.3 | **S1395** | 0.39 |
| **S130** | 0.3 | **S552** | 0.24 | **S974** | 0.25 | **S1396** | 0.27 |
| **S131** | 0.23 | **S553** | 0.34 | **S975** | 0.38 | **S1397** | 0.34 |
| **S132** | 0.26 | **S554** | 0.35 | **S976** | 0.3 | **S1398** | 0.26 |
| **S133** | 0.23 | **S555** | 0.46 | **S977** | 0.22 | **S1399** | 0.3 |
| **S134** | 0.23 | **S556** | 0.3 | **S978** | 0.26 | **S1400** | 0.24 |
| **S135** | 0.28 | **S557** | 0.46 | **S979** | 0.35 | **S1401** | 0.42 |
| **S136** | 0.47 | **S558** | 0.32 | **S980** | 0.23 | **S1402** | 0.29 |
| **S137** | 0.34 | **S559** | 0.25 | **S981** | 0.29 | **S1403** | 0.3 |
| **S138** | 0.51 | **S560** | 0.24 | **S982** | 0.25 | **S1404** | 0.27 |
| **S139** | 0.73 | **S561** | 0.52 | **S983** | 0.38 | **S1405** | 0.45 |
| **S140** | 0.29 | **S562** | 0.21 | **S984** | 0.31 | **S1406** | 0.2 |
| **S141** | 0.42 | **S563** | 0.3 | **S985** | 0.26 | **S1407** | 0.35 |
| **S142** | 0.39 | **S564** | 0.38 | **S986** | 0.47 | **S1408** | 0.28 |
| **S143** | 0.2 | **S565** | 0.25 | **S987** | 0.43 | **S1409** | 0.31 |
| **S144** | 0.27 | **S566** | 0.5 | **S988** | 0.27 | **S1410** | 0.3 |
| **S145** | 0.3 | **S567** | 0.29 | **S989** | 0.22 | **S1411** | 0.3 |
| **S146** | 0.29 | **S568** | 0.64 | **S990** | 0.55 | **S1412** | 0.42 |
| **S147** | 0.24 | **S569** | 0.28 | **S991** | 0.29 | **S1413** | 0.23 |
| **S148** | 0.75 | **S570** | 0.23 | **S992** | 0.3 | **S1414** | 0.27 |
| **S149** | 0.76 | **S571** | 0.22 | **S993** | 0.46 | **S1415** | 0.29 |
| **S150** | 0.24 | **S572** | 0.26 | **S994** | 0.39 | **S1416** | 0.37 |
| **S151** | 0.63 | **S573** | 0.3 | **S995** | 0.43 | **S1417** | 0.25 |
| **S152** | 0.48 | **S574** | 0.23 | **S996** | 0.3 | **S1418** | 0.24 |
| **S153** | 0.81 | **S575** | 0.46 | **S997** | 0.22 | **S1419** | 0.26 |
| **S154** | 0.6 | **S576** | 0.4 | **S998** | 0.29 | **S1420** | 0.54 |
| **S155** | 0.23 | **S577** | 0.26 | **S999** | 0.29 | **S1421** | 0.21 |
| **S156** | 0.33 | **S578** | 0.3 | **S1000** | 0.25 | **S1422** | 0.23 |
| **S157** | 0.26 | **S579** | 0.84 | **S1001** | 0.22 | **S1423** | 0.21 |
| **S158** | 0.23 | **S580** | 0.26 | **S1002** | 0.3 | **S1424** | 0.29 |
| **S159** | 0.71 | **S581** | 0.25 | **S1003** | 0.26 | **S1425** | 0.29 |
| **S160** | 0.8 | **S582** | 0.21 | **S1004** | 0.29 | **S1426** | 0.21 |
| **S161** | 0.33 | **S583** | 0.27 | **S1005** | 0.26 | **S1427** | 0.3 |
| **S162** | 0.29 | **S584** | 0.3 | **S1006** | 0.39 | **S1428** | 0.3 |
| **S163** | 0.33 | **S585** | 0.27 | **S1007** | 0.21 | **S1429** | 0.23 |
| **S164** | 0.34 | **S586** | 0.41 | **S1008** | 0.22 | **S1430** | 0.84 |
| **S165** | 0.28 | **S587** | 0.26 | **S1009** | 0.25 | **S1431** | 0.22 |
| **S166** | 0.27 | **S588** | 0.26 | **S1010** | 0.21 | **S1432** | 0.28 |
| **S167** | 0.3 | **S589** | 0.36 | **S1011** | 0.52 | **S1433** | 0.21 |
| **S168** | 0.24 | **S590** | 0.3 | **S1012** | 0.3 | **S1434** | 0.26 |
| **S169** | 0.21 | **S591** | 0.3 | **S1013** | 0.27 | **S1435** | 0.22 |
| **S170** | 0.67 | **S592** | 0.24 | **S1014** | 0.21 | **S1436** | 0.23 |
| **S171** | 0.36 | **S593** | 0.26 | **S1015** | 0.35 | **S1437** | 0.28 |
| **S172** | 0.21 | **S594** | 0.73 | **S1016** | 0.3 | **S1438** | 0.23 |
| **S173** | 0.22 | **S595** | 0.32 | **S1017** | 0.34 | **S1439** | 0.23 |
| **S174** | 0.36 | **S596** | 0.48 | **S1018** | 0.21 | **S1440** | 0.37 |
| **S175** | 0.21 | **S597** | 0.32 | **S1019** | 0.25 | **S1441** | 0.3 |
| **S176** | 0.61 | **S598** | 0.55 | **S1020** | 0.32 | **S1442** | 0.28 |
| **S177** | 0.25 | **S599** | 0.41 | **S1021** | 0.47 | **S1443** | 0.22 |
| **S178** | 0.27 | **S600** | 0.28 | **S1022** | 0.23 | **S1444** | 0.87 |
| **S179** | 0.25 | **S601** | 0.33 | **S1023** | 0.28 | **S1445** | 0.26 |
| **S180** | 0.27 | **S602** | 0.29 | **S1024** | 0.27 | **S1446** | 0.29 |
| **S181** | 0.3 | **S603** | 0.32 | **S1025** | 0.29 | **S1447** | 0.32 |
| **S182** | 0.21 | **S604** | 0.24 | **S1026** | 0.32 | **S1448** | 0.3 |
| **S183** | 0.54 | **S605** | 0.23 | **S1027** | 0.28 | **S1449** | 0.23 |
| **S184** | 0.94 | **S606** | 0.29 | **S1028** | 0.21 | **S1450** | 0.3 |
| **S185** | 0.27 | **S607** | 0.32 | **S1029** | 0.2 | **S1451** | 0.29 |
| **S186** | 0.88 | **S608** | 0.4 | **S1030** | 0.34 | **S1452** | 0.24 |
| **S187** | 0.28 | **S609** | 0.27 | **S1031** | 0.3 | **S1453** | 0.28 |
| **S188** | 0.3 | **S610** | 0.21 | **S1032** | 0.28 | **S1454** | 0.29 |
| **S189** | 0.4 | **S611** | 0.33 | **S1033** | 0.23 | **S1455** | 0.3 |
| **S190** | 0.29 | **S612** | 0.28 | **S1034** | 0.3 | **S1456** | 0.26 |
| **S191** | 0.26 | **S613** | 0.26 | **S1035** | 0.4 | **S1457** | 0.36 |
| **S192** | 0.27 | **S614** | 0.3 | **S1036** | 0.48 | **S1458** | 0.42 |
| **S193** | 0.28 | **S615** | 0.65 | **S1037** | 0.49 | **S1459** | 0.49 |
| **S194** | 0.28 | **S616** | 0.2 | **S1038** | 0.39 | **S1460** | 0.25 |
| **S195** | 0.26 | **S617** | 0.26 | **S1039** | 0.23 | **S1461** | 0.22 |
| **S196** | 0.28 | **S618** | 0.59 | **S1040** | 0.24 | **S1462** | 0.27 |
| **S197** | 0.23 | **S619** | 0.48 | **S1041** | 0.3 | **S1463** | 0.2 |
| **S198** | 0.28 | **S620** | 0.25 | **S1042** | 0.3 | **S1464** | 0.26 |
| **S199** | 0.39 | **S621** | 0.3 | **S1043** | 0.3 | **S1465** | 0.21 |
| **S200** | 0.27 | **S622** | 0.33 | **S1044** | 0.5 | **S1466** | 0.65 |
| **S201** | 0.25 | **S623** | 0.41 | **S1045** | 0.28 | **S1467** | 0.26 |
| **S202** | 0.27 | **S624** | 0.45 | **S1046** | 0.35 | **S1468** | 0.25 |
| **S203** | 0.61 | **S625** | 0.33 | **S1047** | 0.21 | **S1469** | 0.3 |
| **S204** | 0.29 | **S626** | 0.58 | **S1048** | 0.4 | **S1470** | 0.47 |
| **S205** | 0.27 | **S627** | 0.28 | **S1049** | 0.3 | **S1471** | 0.41 |
| **S206** | 0.35 | **S628** | 0.25 | **S1050** | 0.25 | **S1472** | 0.28 |
| **S207** | 0.25 | **S629** | 0.24 | **S1051** | 0.21 | **S1473** | 0.23 |
| **S208** | 0.87 | **S630** | 0.3 | **S1052** | 0.3 | **S1474** | 0.29 |
| **S209** | 0.27 | **S631** | 0.42 | **S1053** | 0.68 | **S1475** | 0.29 |
| **S210** | 0.21 | **S632** | 0.21 | **S1054** | 0.66 | **S1476** | 0.3 |
| **S211** | 0.34 | **S633** | 0.41 | **S1055** | 0.73 | **S1477** | 0.27 |
| **S212** | 0.27 | **S634** | 0.25 | **S1056** | 0.26 | **S1478** | 0.42 |
| **S213** | 0.3 | **S635** | 0.3 | **S1057** | 0.72 | **S1479** | 0.21 |
| **S214** | 0.27 | **S636** | 0.28 | **S1058** | 0.27 | **S1480** | 0.24 |
| **S215** | 0.29 | **S637** | 0.41 | **S1059** | 0.74 | **S1481** | 0.86 |
| **S216** | 0.35 | **S638** | 0.38 | **S1060** | 0.4 | **S1482** | 0.25 |
| **S217** | 0.54 | **S639** | 0.24 | **S1061** | 0.27 | **S1483** | 0.24 |
| **S218** | 0.42 | **S640** | 0.28 | **S1062** | 0.38 | **S1484** | 0.25 |
| **S219** | 0.31 | **S641** | 0.26 | **S1063** | 0.2 | **S1485** | 0.35 |
| **S220** | 0.44 | **S642** | 0.23 | **S1064** | 0.37 | **S1486** | 0.3 |
| **S221** | 0.33 | **S643** | 0.23 | **S1065** | 0.38 | **S1487** | 0.46 |
| **S222** | 0.63 | **S644** | 0.22 | **S1066** | 0.77 | **S1488** | 0.31 |
| **S223** | 0.21 | **S645** | 0.3 | **S1067** | 0.3 | **S1489** | 0.29 |
| **S224** | 0.28 | **S646** | 0.72 | **S1068** | 0.23 | **S1490** | 0.3 |
| **S225** | 0.25 | **S647** | 0.26 | **S1069** | 0.28 | **S1491** | 0.2 |
| **S226** | 0.25 | **S648** | 0.38 | **S1070** | 0.27 | **S1492** | 0.2 |
| **S227** | 0.29 | **S649** | 0.36 | **S1071** | 0.2 | **S1493** | 0.24 |
| **S228** | 0.54 | **S650** | 0.65 | **S1072** | 0.51 | **S1494** | 0.28 |
| **S229** | 0.29 | **S651** | 0.23 | **S1073** | 0.3 | **S1495** | 0.26 |
| **S230** | 0.21 | **S652** | 0.29 | **S1074** | 0.54 | **S1496** | 0.69 |
| **S231** | 0.64 | **S653** | 0.22 | **S1075** | 0.62 | **S1497** | 0.3 |
| **S232** | 0.26 | **S654** | 0.32 | **S1076** | 0.25 | **S1498** | 0.26 |
| **S233** | 0.77 | **S655** | 0.8 | **S1077** | 0.51 | **S1499** | 0.59 |
| **S234** | 0.28 | **S656** | 0.32 | **S1078** | 0.3 | **S1500** | 0.3 |
| **S235** | 0.21 | **S657** | 0.3 | **S1079** | 0.25 | **S1501** | 0.3 |
| **S236** | 0.26 | **S658** | 0.78 | **S1080** | 0.29 | **S1502** | 0.28 |
| **S237** | 0.58 | **S659** | 0.22 | **S1081** | 0.28 | **S1503** | 0.26 |
| **S238** | 0.22 | **S660** | 0.79 | **S1082** | 0.35 | **S1504** | 0.3 |
| **S239** | 0.58 | **S661** | 0.59 | **S1083** | 0.36 | **S1505** | 0.29 |
| **S240** | 0.28 | **S662** | 0.23 | **S1084** | 0.24 | **S1506** | 0.3 |
| **S241** | 0.53 | **S663** | 0.71 | **S1085** | 0.26 | **S1507** | 0.27 |
| **S242** | 0.28 | **S664** | 0.43 | **S1086** | 0.46 | **S1508** | 0.23 |
| **S243** | 0.27 | **S665** | 0.27 | **S1087** | 0.21 | **S1509** | 0.35 |
| **S244** | 0.63 | **S666** | 0.28 | **S1088** | 0.34 | **S1510** | 0.3 |
| **S245** | 0.45 | **S667** | 0.23 | **S1089** | 0.29 | **S1511** | 0.26 |
| **S246** | 0.51 | **S668** | 0.3 | **S1090** | 0.39 | **S1512** | 0.26 |
| **S247** | 0.21 | **S669** | 0.25 | **S1091** | 0.59 | **S1513** | 0.25 |
| **S248** | 0.34 | **S670** | 0.48 | **S1092** | 0.3 | **S1514** | 0.24 |
| **S249** | 0.54 | **S671** | 0.27 | **S1093** | 0.27 | **S1515** | 0.22 |
| **S250** | 0.38 | **S672** | 0.2 | **S1094** | 0.22 | **S1516** | 0.27 |
| **S251** | 0.56 | **S673** | 0.25 | **S1095** | 0.26 | **S1517** | 0.31 |
| **S252** | 0.33 | **S674** | 0.48 | **S1096** | 0.23 | **S1518** | 0.34 |
| **S253** | 0.26 | **S675** | 0.43 | **S1097** | 0.29 | **S1519** | 0.28 |
| **S254** | 0.77 | **S676** | 0.27 | **S1098** | 0.23 | **S1520** | 0.28 |
| **S255** | 0.4 | **S677** | 0.36 | **S1099** | 0.54 | **S1521** | 0.22 |
| **S256** | 0.28 | **S678** | 0.31 | **S1100** | 0.5 | **S1522** | 0.28 |
| **S257** | 0.3 | **S679** | 0.3 | **S1101** | 0.34 | **S1523** | 0.23 |
| **S258** | 0.43 | **S680** | 0.23 | **S1102** | 0.28 | **S1524** | 0.26 |
| **S259** | 0.43 | **S681** | 0.22 | **S1103** | 0.26 | **S1525** | 0.21 |
| **S260** | 0.49 | **S682** | 0.23 | **S1104** | 0.22 | **S1526** | 0.28 |
| **S261** | 0.48 | **S683** | 0.25 | **S1105** | 0.33 | **S1527** | 0.24 |
| **S262** | 0.3 | **S684** | 0.3 | **S1106** | 0.29 | **S1528** | 0.26 |
| **S263** | 0.78 | **S685** | 0.24 | **S1107** | 0.58 | **S1529** | 0.28 |
| **S264** | 0.36 | **S686** | 0.26 | **S1108** | 0.21 | **S1530** | 0.27 |
| **S265** | 0.28 | **S687** | 0.28 | **S1109** | 0.3 | **S1531** | 0.4 |
| **S266** | 0.3 | **S688** | 0.4 | **S1110** | 0.32 | **S1532** | 0.3 |
| **S267** | 0.3 | **S689** | 0.4 | **S1111** | 0.27 | **S1533** | 0.27 |
| **S268** | 0.41 | **S690** | 0.35 | **S1112** | 0.44 | **S1534** | 0.24 |
| **S269** | 0.3 | **S691** | 0.23 | **S1113** | 0.26 | **S1535** | 0.22 |
| **S270** | 0.22 | **S692** | 0.3 | **S1114** | 0.25 | **S1536** | 0.55 |
| **S271** | 0.2 | **S693** | 0.35 | **S1115** | 0.22 | **S1537** | 0.23 |
| **S272** | 0.45 | **S694** | 0.66 | **S1116** | 0.25 | **S1538** | 0.24 |
| **S273** | 0.34 | **S695** | 0.64 | **S1117** | 0.21 | **S1539** | 0.28 |
| **S274** | 0.29 | **S696** | 0.61 | **S1118** | 0.24 | **S1540** | 0.29 |
| **S275** | 0.21 | **S697** | 0.23 | **S1119** | 0.29 | **S1541** | 0.26 |
| **S276** | 0.24 | **S698** | 0.23 | **S1120** | 0.27 | **S1542** | 0.3 |
| **S277** | 0.39 | **S699** | 0.81 | **S1121** | 0.22 | **S1543** | 0.44 |
| **S278** | 0.28 | **S700** | 0.27 | **S1122** | 0.27 | **S1544** | 0.27 |
| **S279** | 0.26 | **S701** | 0.36 | **S1123** | 0.34 | **S1545** | 0.26 |
| **S280** | 0.25 | **S702** | 0.37 | **S1124** | 0.4 | **S1546** | 0.3 |
| **S281** | 0.24 | **S703** | 0.43 | **S1125** | 0.46 | **S1547** | 0.25 |
| **S282** | 0.27 | **S704** | 0.37 | **S1126** | 0.32 | **S1548** | 0.28 |
| **S283** | 0.29 | **S705** | 0.23 | **S1127** | 0.55 | **S1549** | 0.25 |
| **S284** | 0.31 | **S706** | 0.35 | **S1128** | 0.76 | **S1550** | 0.22 |
| **S285** | 0.3 | **S707** | 0.21 | **S1129** | 0.2 | **S1551** | 0.2 |
| **S286** | 0.6 | **S708** | 0.25 | **S1130** | 0.72 | **S1552** | 0.66 |
| **S287** | 0.3 | **S709** | 0.47 | **S1131** | 0.22 | **S1553** | 0.3 |
| **S288** | 0.27 | **S710** | 0.78 | **S1132** | 0.63 | **S1554** | 0.22 |
| **S289** | 0.29 | **S711** | 0.31 | **S1133** | 0.3 | **S1555** | 0.2 |
| **S290** | 0.22 | **S712** | 0.21 | **S1134** | 0.3 | **S1556** | 0.24 |
| **S291** | 0.28 | **S713** | 0.23 | **S1135** | 0.27 | **S1557** | 0.28 |
| **S292** | 0.31 | **S714** | 0.21 | **S1136** | 0.47 | **S1558** | 0.3 |
| **S293** | 0.25 | **S715** | 0.95 | **S1137** | 0.3 | **S1559** | 0.24 |
| **S294** | 0.35 | **S716** | 0.45 | **S1138** | 0.27 | **S1560** | 0.28 |
| **S295** | 0.2 | **S717** | 0.25 | **S1139** | 0.28 | **S1561** | 0.22 |
| **S296** | 0.3 | **S718** | 0.23 | **S1140** | 0.3 | **S1562** | 0.3 |
| **S297** | 0.5 | **S719** | 0.21 | **S1141** | 0.28 | **S1563** | 0.29 |
| **S298** | 0.2 | **S720** | 0.33 | **S1142** | 0.3 | **S1564** | 0.3 |
| **S299** | 0.21 | **S721** | 0.3 | **S1143** | 0.33 | **S1565** | 0.3 |
| **S300** | 0.22 | **S722** | 0.26 | **S1144** | 0.54 | **S1566** | 0.48 |
| **S301** | 0.3 | **S723** | 0.22 | **S1145** | 0.46 | **S1567** | 0.2 |
| **S302** | 0.28 | **S724** | 0.22 | **S1146** | 0.34 | **S1568** | 0.3 |
| **S303** | 0.39 | **S725** | 0.23 | **S1147** | 0.23 | **S1569** | 0.23 |
| **S304** | 0.33 | **S726** | 0.33 | **S1148** | 0.25 | **S1570** | 0.22 |
| **S305** | 0.25 | **S727** | 0.3 | **S1149** | 0.3 | **S1571** | 0.37 |
| **S306** | 0.34 | **S728** | 0.31 | **S1150** | 0.22 | **S1572** | 0.22 |
| **S307** | 0.3 | **S729** | 0.26 | **S1151** | 0.39 | **S1573** | 0.21 |
| **S308** | 0.29 | **S730** | 0.27 | **S1152** | 0.52 | **S1574** | 0.3 |
| **S309** | 0.34 | **S731** | 0.42 | **S1153** | 0.35 | **S1575** | 0.22 |
| **S310** | 0.87 | **S732** | 0.62 | **S1154** | 0.25 | **S1576** | 0.45 |
| **S311** | 0.64 | **S733** | 0.25 | **S1155** | 0.25 | **S1577** | 0.3 |
| **S312** | 0.27 | **S734** | 0.78 | **S1156** | 0.57 | **S1578** | 0.31 |
| **S313** | 0.33 | **S735** | 0.4 | **S1157** | 0.36 | **S1579** | 0.39 |
| **S314** | 0.24 | **S736** | 0.27 | **S1158** | 0.53 | **S1580** | 0.93 |
| **S315** | 0.22 | **S737** | 0.3 | **S1159** | 0.3 | **S1581** | 0.88 |
| **S316** | 0.36 | **S738** | 0.27 | **S1160** | 0.26 | **S1582** | 0.91 |
| **S317** | 0.37 | **S739** | 0.3 | **S1161** | 0.29 | **S1583** | 0.22 |
| **S318** | 0.26 | **S740** | 0.22 | **S1162** | 0.4 | **S1584** | 0.3 |
| **S319** | 1 | **S741** | 0.29 | **S1163** | 0.42 | **S1585** | 0.23 |
| **S320** | 0.27 | **S742** | 0.3 | **S1164** | 0.21 | **S1586** | 0.23 |
| **S321** | 0.27 | **S743** | 0.3 | **S1165** | 0.29 | **S1587** | 0.25 |
| **S322** | 0.32 | **S744** | 0.3 | **S1166** | 0.3 | **S1588** | 0.28 |
| **S323** | 0.34 | **S745** | 0.29 | **S1167** | 0.3 | **S1589** | 0.22 |
| **S324** | 0.69 | **S746** | 0.24 | **S1168** | 0.22 | **S1590** | 0.23 |
| **S325** | 0.29 | **S747** | 0.39 | **S1169** | 0.28 | **S1591** | 0.3 |
| **S326** | 0.39 | **S748** | 0.21 | **S1170** | 0.3 | **S1592** | 0.26 |
| **S327** | 0.92 | **S749** | 0.26 | **S1171** | 0.22 | **S1593** | 0.3 |
| **S328** | 0.37 | **S750** | 0.26 | **S1172** | 0.44 | **S1594** | 0.24 |
| **S329** | 0.28 | **S751** | 0.26 | **S1173** | 0.27 | **S1595** | 0.23 |
| **S330** | 0.47 | **S752** | 0.55 | **S1174** | 0.23 | **S1596** | 0.56 |
| **S331** | 0.53 | **S753** | 0.24 | **S1175** | 0.4 | **S1597** | 0.21 |
| **S332** | 0.47 | **S754** | 0.26 | **S1176** | 0.25 | **S1598** | 0.3 |
| **S333** | 0.37 | **S755** | 0.25 | **S1177** | 0.26 | **S1599** | 0.21 |
| **S334** | 0.23 | **S756** | 0.21 | **S1178** | 0.2 | **S1600** | 0.3 |
| **S335** | 0.26 | **S757** | 0.46 | **S1179** | 0.22 | **S1601** | 0.25 |
| **S336** | 0.33 | **S758** | 0.4 | **S1180** | 0.3 | **S1602** | 0.27 |
| **S337** | 0.49 | **S759** | 0.26 | **S1181** | 0.29 | **S1603** | 0.2 |
| **S338** | 0.32 | **S760** | 0.28 | **S1182** | 0.33 | **S1604** | 0.25 |
| **S339** | 0.29 | **S761** | 0.21 | **S1183** | 0.25 | **S1605** | 0.48 |
| **S340** | 0.44 | **S762** | 0.27 | **S1184** | 0.29 | **S1606** | 0.24 |
| **S341** | 0.3 | **S763** | 0.23 | **S1185** | 0.39 | **S1607** | 0.3 |
| **S342** | 0.24 | **S764** | 0.34 | **S1186** | 0.3 | **S1608** | 0.59 |
| **S343** | 0.3 | **S765** | 0.23 | **S1187** | 0.33 | **S1609** | 0.29 |
| **S344** | 0.3 | **S766** | 0.22 | **S1188** | 0.32 | **S1610** | 0.95 |
| **S345** | 0.26 | **S767** | 0.39 | **S1189** | 0.3 | **S1611** | 0.23 |
| **S346** | 0.32 | **S768** | 0.3 | **S1190** | 0.55 | **S1612** | 0.86 |
| **S347** | 0.27 | **S769** | 0.62 | **S1191** | 0.24 | **S1613** | 0.43 |
| **S348** | 0.33 | **S770** | 0.23 | **S1192** | 0.3 | **S1614** | 0.52 |
| **S349** | 0.28 | **S771** | 0.52 | **S1193** | 0.34 | **S1615** | 0.26 |
| **S350** | 0.28 | **S772** | 0.55 | **S1194** | 0.28 | **S1616** | 0.3 |
| **S351** | 0.3 | **S773** | 0.23 | **S1195** | 0.69 | **S1617** | 0.49 |
| **S352** | 0.29 | **S774** | 0.31 | **S1196** | 0.25 | **S1618** | 0.21 |
| **S353** | 0.32 | **S775** | 0.24 | **S1197** | 0.3 | **S1619** | 0.34 |
| **S354** | 0.26 | **S776** | 0.23 | **S1198** | 0.28 | **S1620** | 0.25 |
| **S355** | 0.24 | **S777** | 0.3 | **S1199** | 0.34 | **S1621** | 0.3 |
| **S356** | 0.3 | **S778** | 0.41 | **S1200** | 0.26 | **S1622** | 0.3 |
| **S357** | 0.27 | **S779** | 0.27 | **S1201** | 0.24 | **S1623** | 0.21 |
| **S358** | 0.25 | **S780** | 0.39 | **S1202** | 0.39 | **S1624** | 0.3 |
| **S359** | 0.23 | **S781** | 0.44 | **S1203** | 0.22 | **S1625** | 0.29 |
| **S360** | 0.52 | **S782** | 0.39 | **S1204** | 0.58 | **S1626** | 0.27 |
| **S361** | 0.22 | **S783** | 0.26 | **S1205** | 0.3 | **S1627** | 0.3 |
| **S362** | 0.42 | **S784** | 0.34 | **S1206** | 0.32 | **S1628** | 0.3 |
| **S363** | 0.22 | **S785** | 0.3 | **S1207** | 0.29 | **S1629** | 0.3 |
| **S364** | 0.28 | **S786** | 0.3 | **S1208** | 0.24 | **S1630** | 0.24 |
| **S365** | 0.3 | **S787** | 0.34 | **S1209** | 0.31 | **S1631** | 0.3 |
| **S366** | 0.2 | **S788** | 0.3 | **S1210** | 0.54 | **S1632** | 0.22 |
| **S367** | 0.72 | **S789** | 0.77 | **S1211** | 0.26 | **S1633** | 0.25 |
| **S368** | 0.2 | **S790** | 0.36 | **S1212** | 0.43 | **S1634** | 0.35 |
| **S369** | 0.24 | **S791** | 0.23 | **S1213** | 0.26 | **S1635** | 0.27 |
| **S370** | 0.22 | **S792** | 0.21 | **S1214** | 0.52 | **S1636** | 0.32 |
| **S371** | 0.23 | **S793** | 0.28 | **S1215** | 0.48 | **S1637** | 0.27 |
| **S372** | 0.27 | **S794** | 0.4 | **S1216** | 0.3 | **S1638** | 0.25 |
| **S373** | 0.34 | **S795** | 0.23 | **S1217** | 0.3 | **S1639** | 0.45 |
| **S374** | 0.32 | **S796** | 0.23 | **S1218** | 0.52 | **S1640** | 0.3 |
| **S375** | 0.31 | **S797** | 0.22 | **S1219** | 0.37 | **S1641** | 0.3 |
| **S376** | 0.3 | **S798** | 0.47 | **S1220** | 0.23 | **S1642** | 0.2 |
| **S377** | 0.21 | **S799** | 0.33 | **S1221** | 0.3 | **S1643** | 0.51 |
| **S378** | 0.31 | **S800** | 0.25 | **S1222** | 0.32 | **S1644** | 0.48 |
| **S379** | 0.22 | **S801** | 0.3 | **S1223** | 0.25 | **S1645** | 0.29 |
| **S380** | 0.27 | **S802** | 0.27 | **S1224** | 0.41 | **S1646** | 0.3 |
| **S381** | 0.28 | **S803** | 0.49 | **S1225** | 0.3 | **S1647** | 0.35 |
| **S382** | 0.37 | **S804** | 0.23 | **S1226** | 0.3 | **S1648** | 0.38 |
| **S383** | 0.27 | **S805** | 0.28 | **S1227** | 0.27 | **S1649** | 0.34 |
| **S384** | 0.29 | **S806** | 0.42 | **S1228** | 0.26 | **S1650** | 0.29 |
| **S385** | 0.28 | **S807** | 0.24 | **S1229** | 0.29 | **S1651** | 0.26 |
| **S386** | 0.72 | **S808** | 0.3 | **S1230** | 0.64 | **S1652** | 0.38 |
| **S387** | 0.3 | **S809** | 0.27 | **S1231** | 0.3 | **S1653** | 0.26 |
| **S388** | 0.82 | **S810** | 0.24 | **S1232** | 0.32 | **S1654** | 0.26 |
| **S389** | 0.3 | **S811** | 0.3 | **S1233** | 0.26 | **S1655** | 0.23 |
| **S390** | 0.7 | **S812** | 0.71 | **S1234** | 0.84 | **S1656** | 0.21 |
| **S391** | 0.2 | **S813** | 0.3 | **S1235** | 0.23 | **S1657** | 0.3 |
| **S392** | 0.3 | **S814** | 0.24 | **S1236** | 0.35 | **S1658** | 0.23 |
| **S393** | 0.46 | **S815** | 0.24 | **S1237** | 0.31 | **S1659** | 0.22 |
| **S394** | 0.24 | **S816** | 0.37 | **S1238** | 0.28 | **S1660** | 0.26 |
| **S395** | 0.39 | **S817** | 0.6 | **S1239** | 0.37 | **S1661** | 0.25 |
| **S396** | 0.3 | **S818** | 0.67 | **S1240** | 0.3 | **S1662** | 0.22 |
| **S397** | 0.31 | **S819** | 0.3 | **S1241** | 0.46 | **S1663** | 0.24 |
| **S398** | 0.35 | **S820** | 0.69 | **S1242** | 0.28 | **S1664** | 0.3 |
| **S399** | 0.28 | **S821** | 0.47 | **S1243** | 0.24 | **S1665** | 0.25 |
| **S400** | 0.3 | **S822** | 0.3 | **S1244** | 0.34 | **S1666** | 0.29 |
| **S401** | 0.27 | **S823** | 0.24 | **S1245** | 0.29 | **S1667** | 0.27 |
| **S402** | 0.27 | **S824** | 0.3 | **S1246** | 0.27 | **S1668** | 0.3 |
| **S403** | 0.22 | **S825** | 0.3 | **S1247** | 0.32 | **S1669** | 0.26 |
| **S404** | 0.23 | **S826** | 0.29 | **S1248** | 0.29 | **S1670** | 0.25 |
| **S405** | 0.36 | **S827** | 0.22 | **S1249** | 0.36 | **S1671** | 0.25 |
| **S406** | 0.28 | **S828** | 0.3 | **S1250** | 0.28 | **S1672** | 0.61 |
| **S407** | 0.33 | **S829** | 0.26 | **S1251** | 0.27 | **S1673** | 0.45 |
| **S408** | 0.22 | **S830** | 0.29 | **S1252** | 0.64 | **S1674** | 0.95 |
| **S409** | 0.24 | **S831** | 0.26 | **S1253** | 0.48 | **S1675** | 0.26 |
| **S410** | 0.21 | **S832** | 0.22 | **S1254** | 0.27 | **S1676** | 0.34 |
| **S411** | 0.52 | **S833** | 0.56 | **S1255** | 0.62 | **S1677** | 0.3 |
| **S412** | 0.29 | **S834** | 0.28 | **S1256** | 0.3 | **S1678** | 0.22 |
| **S413** | 0.33 | **S835** | 0.7 | **S1257** | 0.27 | **S1679** | 0.25 |
| **S414** | 0.21 | **S836** | 0.27 | **S1258** | 0.25 | **S1680** | 0.27 |
| **S415** | 0.3 | **S837** | 0.28 | **S1259** | 0.24 | **S1681** | 0.24 |
| **S416** | 0.56 | **S838** | 0.27 | **S1260** | 0.26 | **S1682** | 0.27 |
| **S417** | 0.59 | **S839** | 0.3 | **S1261** | 0.3 | **S1683** | 0.22 |
| **S418** | 0.45 | **S840** | 0.26 | **S1262** | 0.29 | **S1684** | 0.21 |
| **S419** | 0.3 | **S841** | 0.28 | **S1263** | 0.3 | **S1685** | 0.25 |
| **S420** | 0.61 | **S842** | 0.3 | **S1264** | 0.3 | **S1686** | 0.3 |
| **S421** | 0.31 | **S843** | 0.35 | **S1265** | 0.53 | **S1687** | 0.42 |
| **S422** | 0.25 | **S844** | 0.56 | **S1266** | 0.3 | **S1688** | 0.22 |
